# A 5.8 GHz 1.8 V +20 dBm 32.5% PAE Power Amplifier for a Short-Range Over-the-Air WPT Application

**DOI:** 10.3390/s23115279

**Published:** 2023-06-02

**Authors:** Myonggwan Kim, Reza E. Rad, Sungjin Kim, Younggun Pu, Yeonjae Jung, Hyungki Huh, Joonmo Yoo, Seokkee Kim, Kangyoon Lee

**Affiliations:** 1 Department of Electrical and Computer Engineering, Sungkyunkwan University, Suwon 16419, Republic of Korea; alex819@g.skku.edu (M.K.); sun107ksj@skku.edu (S.K.); hara1015@skku.edu (Y.P.); yj.jung@skku.edu (Y.J.); gray.huh@skku.edu (H.H.); fiance2@skku.edu (J.Y.); seokkeekim@skku.edu (S.K.); 2 SKAIChips Co., Ltd., Suwon 16419, Republic of Korea; reza@skku.edu

**Keywords:** over-the-air wireless power transfer (WPT), custom-made transformer, power amplifier, 5.8 GHz

## Abstract

This paper presents a 5.8 GHz differential cascode power amplifier for an over-the-air wireless power transfer application. Over-the-air wireless power transfer provides a variety of benefits in several applications such as the Internet of Things and medical implantation applications. The proposed PA features two fully differentially active stages with a custom-designed transformer to provide a single-ended output. The custom-made transformer shows a high quality factor, as high as 11.6 and 11.2 for the primary and secondary sides at 5.8 GHz. Fabricated using a standard 180 nm CMOS process, the amplifier achieves input and output matching of −14.7 dB and −29.7 dB, respectively. To achieve a high power level and efficiency, accurate optimization through power matching, Power Added Efficiency (PAE), and the design of the transformer are carried out while the supply voltage is limited to 1.8 V. Measurement results show a 20 dBm output power with a PAE as high as 32.5%, which makes the PA suitable for application, and it can be implanted while arrayed with various antenna arrays. Finally, a FOM is introduced to compare the performance of the work with similar works in the literature.

## 1. Introduction

With the increase in the Internet of Things (IoT), smart homes, medical implantation, and similar other applications in recent years, the need for fast and convenient power charging is increased, along with a rapid increase in the number of mobile devices and the growing demand for mobile Internet of Things (IoT) applications. The idea of WPT has researched for many years [1,2,3,4]. WPT is a practical method for the wireless charging of the implanted devices in the body for medical applications, and several studies have been conducted on biomedical wireless charging and data communication [5,6,7,8,9,10]. The most interesting power charging method in the last decade has been wireless charging. In [1], a Wireless Power Transfer (WPT) charger system is introduced that provides 15-watt changeability for the charging device with three different standards. The work provides a possibility for charging through charging coils, and the power amplifier structure is designed to provide mid-range power for this purpose. The issue of these works is that the charging device must be placed exactly on the coil to be charged, which is not suitable for our desired applications.

The most recent field of research for WPT is Over-The-Air (OTA) WPT systems. The idea is to transmit radio frequency (RF) signals with a high enough power level to the charging target devices through a distance path without contacting the coils. A short-range OTA WPT system targets the devices around 1 m but has less complexity in terms of the required specifications and architecture. In [11,12], an OTA WPT system is proposed with a power transmitter unit. This work is formed by the combination of artificial intelligence (AI) and the enhancement of phase-adjustable power transmitters. The idea is to detect the location of the target (the device that is to be charged at a distance) and changing the phases of the power-unit arrays through AI-based positioning and phase control algorithms [11].

In this paper, a power amplifier for a short-range OTA WPT is proposed. The work operates with a low power supply, and several techniques are used to bring about a higher efficiency.

## 2. Proposed Power-Amplifier Structure

**Figure 1:**
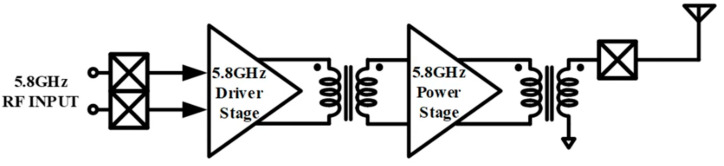
Top Block Diagram of the WPT Transmitter.

One critical concern in enhancing PAs is managing the voltage tension over the transistors. In addition to the power capability, the other requirement is a low cost. This is a concern with high-volume products for consumer WPT devices and has resulted in a comeback path for CMOS devices, while GaAs are used for high-GHz military and satellite applications for communications, which are expensive. Figure 1 shows a block diagram of the proposed WPT PA, which is formed by a driver stage, an interstage balun, a power stage, and finally an output balun. The driver stage must have a low-input capacitance in order to not have a high loading effect on the Voltage-Controlled Oscillator (VCO) which is placed before the PA in the transmitter chain. Therefore, the designs of the interstage and output baluns are critical to obtain the required quality factor (Q) and to provide the required inductances at their primary and secondary sides, which are used for power matching with the corresponding parallel matching capacitances for each of them.

**Figure 2:**
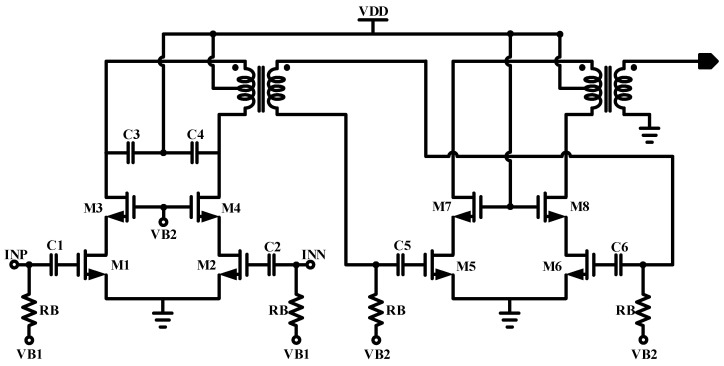
Circuit schematic of differential cascode PA.

Figure 2 illustrates the circuit-level implementation of the proposed PA. Both the driver and the power stages are formed by two differential cascode common source configurations. The input transistor pairs of the driver (M1-M2) are designed to be small enough to not disturb the VCO’s operation that will drive the PA. The cascode pairs M3-M4 and M7-M8 are stacked with common-source M1-M2 and M5-M6, respectively. These cascode transistors enhance the higher power handling for the power amplifier while the large signal swing is divided by two stacked transistor drains to sources. C1-C2 and C5-C6 are the coupling capacitors, while C3-C4 are used to adjust the peak voltage at the secondary side of the transformer at the 5.8 GHz center frequency.

To enhance the CMOS PAs for higher power levels, stacking the transistors is an effective solution. By stacking the transistors while they have a small breakdown voltage compared with the GaAs process, the voltage tension around them is reduced as shown below:

(1) $$V_{\mathrm{ds}}=\frac{V_{\mathrm{Peak}}}{NS}<V_{\mathrm{BD}}$$

where *V*_BD_, *V*_Peak_, and NS are the breakdown voltages of the transistor, the peak voltage of the output voltage, and the number of stacked transistors, respectively. Here, the number of stacked transistors is two, which results in a 50% voltage tension depression caused by the peak output voltage over each transistor. 

The first stage of the proposed two-stage PA serves as the driver stage and is designed with relatively smaller transistors compared to the second stage. Another custom-made transformer is placed between the two stages to isolate the biasing. The input transistors of the driver stage (M1-M2) are chosen to be small enough to prevent a loading effect on the previous stage of the PA. The power stage of the power amplifier is designed with a larger transistor, and the cascode bias of M7-M8 is VDD to provide the maximum power capability for the power stage.

The key to achieving a good power-added efficiency (PAE) for the PA is to provide higher output power levels with lower current consumption. Therefore, selecting appropriate bias voltages of the input transistor pairs of the stages (VB1 and VB2) is critical. The bias level must be chosen to be higher than the threshold voltage and less than the value that does not increase the power. Therefore, the maximum output power and the maximum PAE are obtained with a VB1 of 900 mV, where PAE is the power efficiency and *P*_IN_ is the input power. With more insight into Equation (2), we can conclude a criterion for the PAE of the power amplifier when Equation (2) can be written as follow in watts:

(2) $$\mathrm{PAE}(\%)=\frac{10^{\left(\frac{\left(P_{\mathrm{OUT}\left(\mathrm{dBm}\right)}\right)-30}{10}\right)}\left(W\right)-10^{\left(\frac{\left(P_{\mathrm{IN}\left(\mathrm{dBm}\right)}\right)-30}{10}\right)}\left(W\right)}{P_{\mathrm{DC}}\left(W\right)}\times 100$$

then,

(3) $$P_{\mathrm{DC}}(W)=\frac{10^{\left(\frac{\left(P_{\mathrm{OUT}\left(\mathrm{dBm}\right)}\right)-30}{10}\right)}\left(W\right)-10^{\left(\frac{\left(P_{\mathrm{IN}\left(\mathrm{dBm}\right)}\right)-30}{10}\right)}\left(W\right)}{\mathrm{PAE}\left(\%\right)}\times 100$$

Therefore, using Equations (2) and (3), it is possible to estimate the required output power, power efficiency, and supply power dissipation of PA. Equation (3) is an ideal equation that illustrates the major parameters for enhancing PAE. Although the illustrations do not include any of the realistic parameters of the real world, they demonstrate that to achieve the highest possible PAE, we need to consume a lower level of DC power, or by consuming a fixed amount of it, we enhance higher levels of the power. Therefore, precise DC-biasing is necessary to achieve the maximum available PAE in the real world. In addition, for such an implementation for WPT applications, we do not consider linearity, and the most critical challenge is due to the breakdown voltage restrictions of the CMOS transistors. 

Despite Equation (3) being an ideal relationship, the maximum power is not made easily feasible by adjusting any variable of the equation. For example, the maximum available power of the PA is directly proportional to the value of VDD, which is limited by the breakdown of the transistors. Restricting the VDD to lower values makes it infeasible to reach higher power, and increasing current consumption and *P*_DC_ is useless and decreases the power efficiency. This results in a smaller size, lower input bias level, and lower cascode bias level for the driver stage. However, for the power stage, a different conclusion is reached when the power needs to be maximized using a precise load-pull simulation. A load-pull simulation was conducted for optimized matching, and a matching point was obtained to output the maximum output. An output-matching network is designed based on the load-pull simulation and is placed externally.

**Figure 3:**
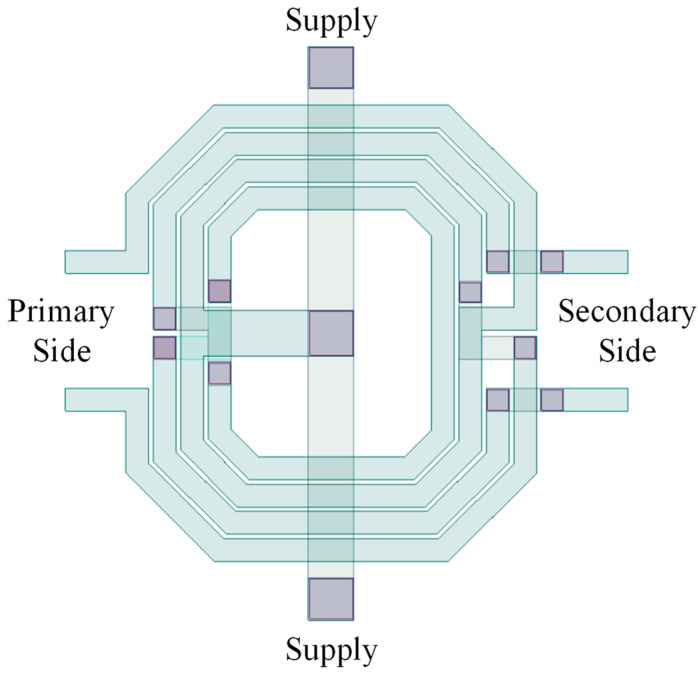
The symmetric custom-made transformer.

**Figure 4:**
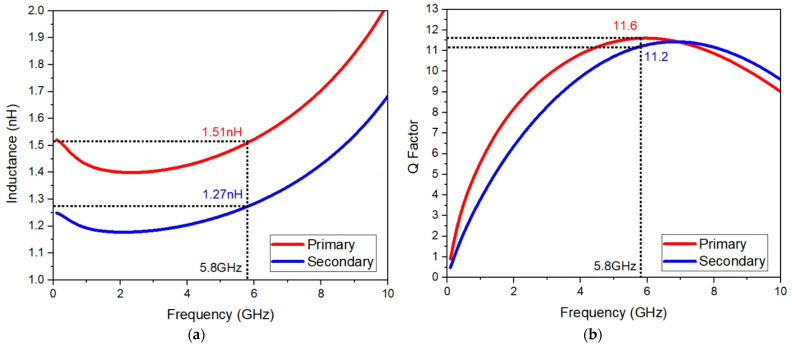
Custom-made transformer of the (**a**) inductance (**b**) Q factor of the transformer.

Both of the transformers used were custom-made and placed between stages and at the output. Figure 3 illustrates the custom-made transformer for PA. The main concern with the integrated transformers is their quality factor and the inductance at the desired center frequency (5.8 GHz). Figure 4a,b show L and Q on the primary and secondary sides, respectively. The primary side has an inductance level of 1.51 nH and a quality factor of 11.6, while the secondary shows an inductance of 1.27 nH and a quality factor of 11.2. 

## 3. Measurement Results

**Figure 5:**
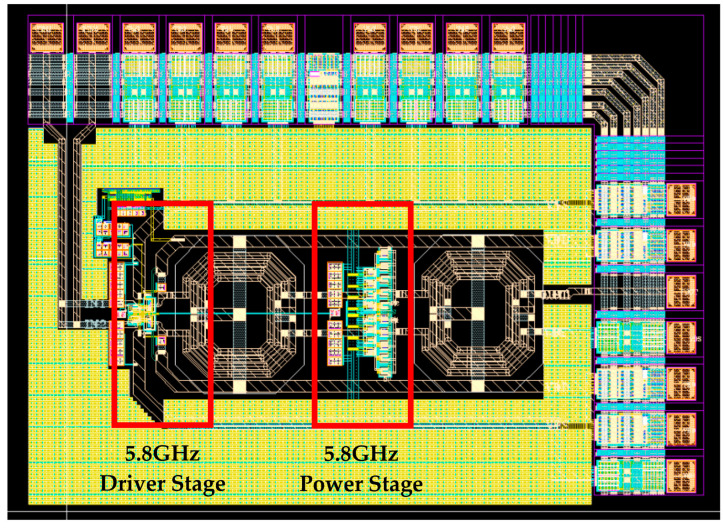
The top layout of the PA.

Figure 5 depicts the top layout of the PA. An inter-stage balun is positioned between the driver stage and the power stage to mitigate signal mismatch. The design of a symmetric arrangement aims to decrease the mismatch on the signal. The input and output signal paths of the driver stage are constructed with the top metal to reduce the effect of the metal layer’s inductance, and it has a wide metal width of 15 um.

High-power PAs have a relatively high current consumption. Therefore, the most critical concern in their physical implementation is using wide enough power routings. Additionally, for the power routings, the top metal is the most suitable choice due to its low sheet resistance. These factors are concerned with the layout implementation of the PA. On the other hand, bounding wires in the packaged ICs must be addressed due to their impact on the overall performance, especially in power matching. Therefore, to minimize the effect of the bounding wires, multiple pads are used for the power liens (grounds and supplies). Finally, supply-to-ground caps are used, which provide a more stable implementation with a lower influence of supply noise.

**Figure 6:**
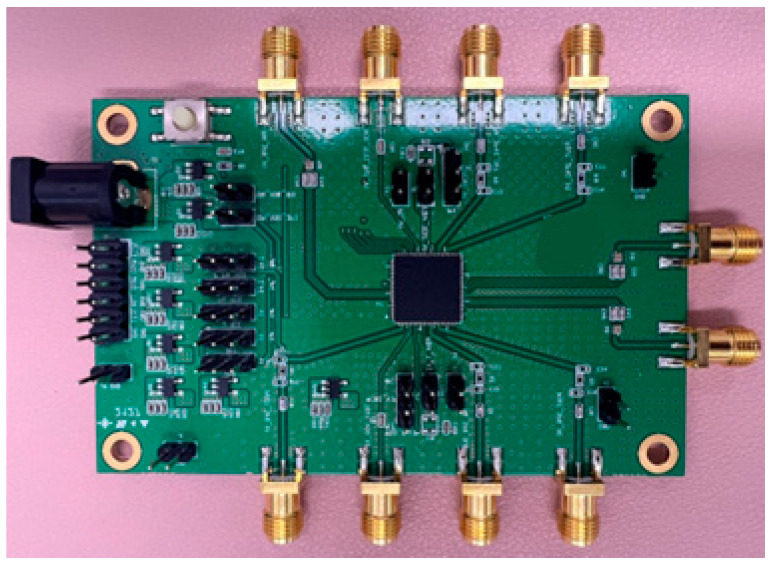
The measurement environment of the sensitivity of the receiver.

**Figure 7:**
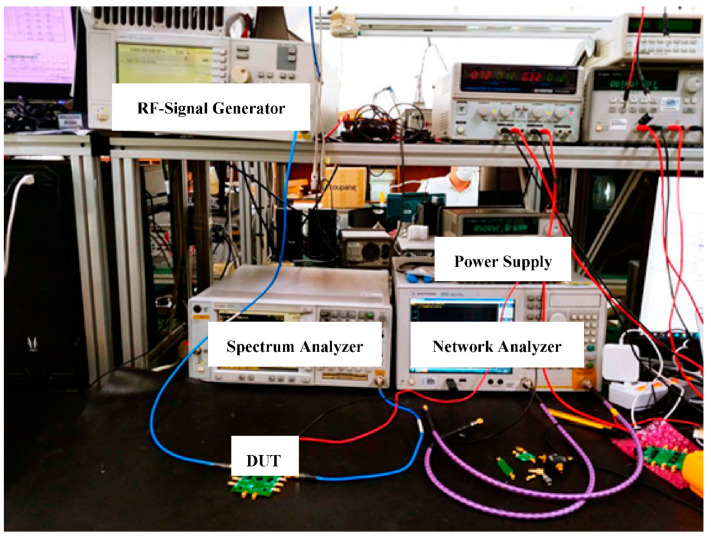
The measurement environment.

The PA is fabricated, and the IC is placed on the test board (Figure 6). The experimental analysis is performed with the measurement setup shown in Figure 7. The measurement setup consists of a power-supply generator, an RF signal generator, a network analyzer, and a spectrum analyzer. The measurement is performed by providing an RF signal at 5.8 GHz using the RF signal generator with 0 dBm power, which is used as the input power for the PA. The output power of the PA is captured using the spectrum analyzer. Additionally, to perform the external matching, a network analyzer is used. The biases and supply voltages are provided using the DC supply generators. 

**Figure 8:**
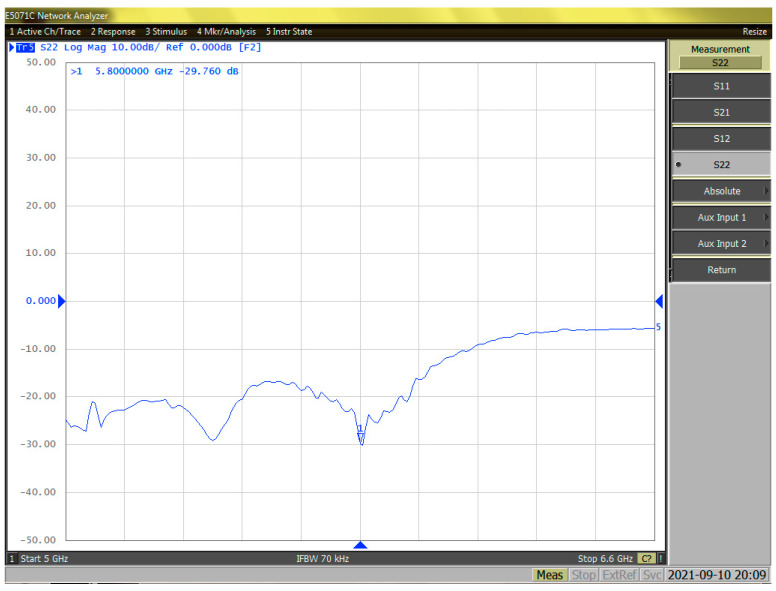
The magnitude of the measured S22 at 5.8 GHz.

**Figure 9:**
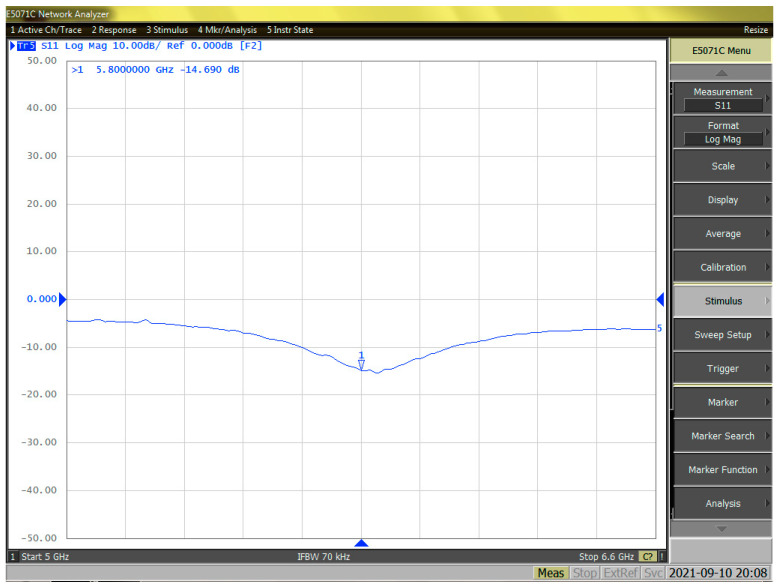
The magnitude of the measured S11 at 5.8 GHz.

The output matching of the PA is performed, and Figure 8 shows S22 in both the Smith chart and the magnitude in dB. Figure 9 shows the measured S11 at 5.8 GHz on the Smith chart and in magnitude (dB). As a result of the measurement, S11 was found to be −14.69 dB and S22 was −29.7 dB. S22, which has a great influence on the output matching, is an important part of the parameters of PA. The high level of −29.7 dB for S22 is due to the custom-made balun, designed based on the current PA Core. This high level of S22 results in a high S22 value that is difficult to implement in inductors provided by conventional processes, and it minimizes the loss of output power and shows high efficiency by reducing losses.

**Figure 10:**
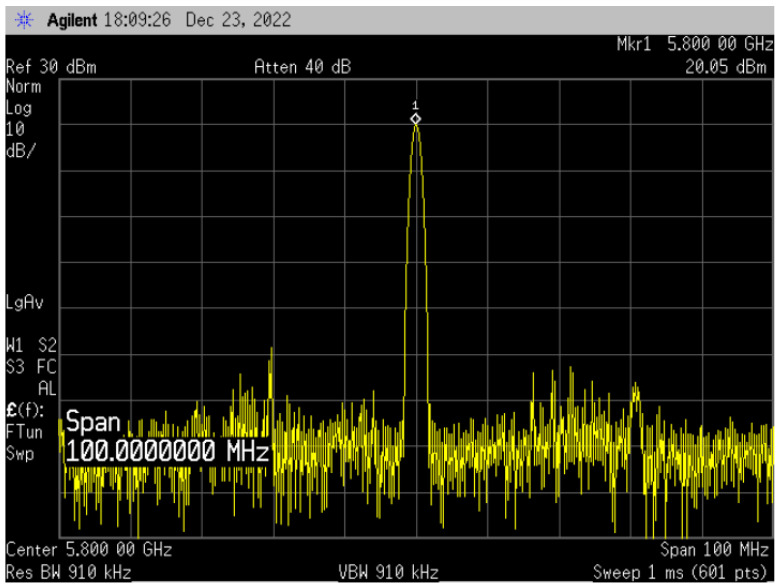
Output Power Measurement Result of Spectrum at 5.8 GHz.

**Figure 11:**
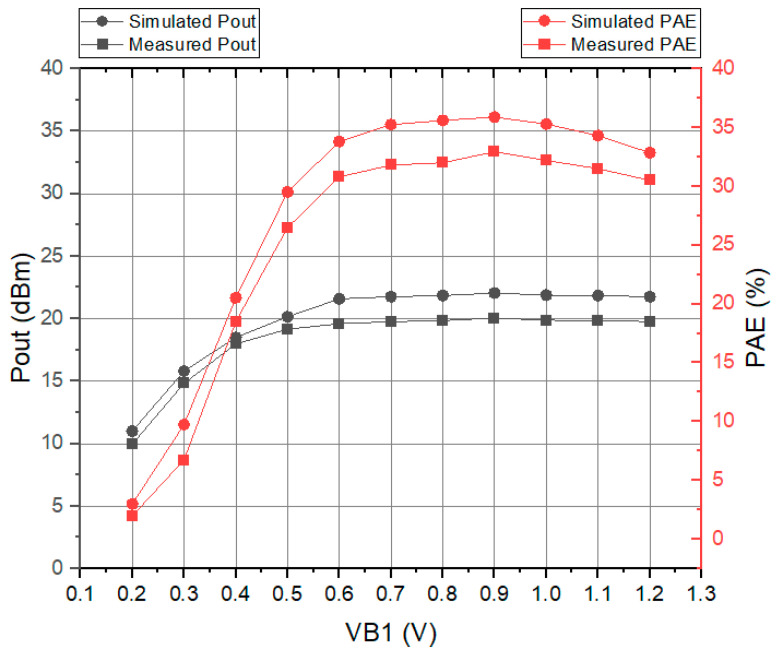
Output power and PAE versus VB1.

Figure 10 illustrates the measurement results of the output power at 5.8 GHz using a spectrum analyzer with an input power of 0 dBm using a RF signal generator. The output power was measured to be 20.05 dBm, indicating a gain of 20 dB. Figure 11 shows the results of simulation and measurement of the output power and PAE according to VB1. When VB1 was biased at 0.9 V, it showed the highest efficiency, and the output power was also the highest. As a result of the measurement, as mentioned above, the output power showed a level of 20.05 dBm, and the PAE showed a level of 32.5%. The calculated FOM using Equation (4) yields a value of 250.43.

On the other hand, Figure 11 reflects the operating points of the PA, which is directly proportional to the bias voltage (VB1). While the output power level is saturated after VB1 = 0.6 V, the PAE is still changing, which is due to the PA’s mode of operation. Therefore, the biasing of the PA is adjusted due to the maximum PAE level.

Table 1 presents a performance summary of the proposed PA and a performance comparison with similar works. To obtain an intuitive analysis of the performance of the PA compared with similar works in the literature, it is necessary to introduce a Figure of Merit (FOM). The FOM must consider the challenging key parameters of the Pas in considering the latest fabrication processes and applications, such as the output power (POUT), the power gain (G), the operating frequency (fc), and the power-added efficiency (PAE). Unfortunately, linearity strongly depends on the operating class of the amplifiers, making it difficult to compare amplifiers of different classes. Therefore, our proposed FOM considers only the four most important parameters: output power, power gain, frequency, and power added efficiency. We propose the following logarithmic representation, with power in dBm, gain in dB, fc in GHz, and PAE (absolute), for benchmarking power amplifiers. Therefore, the Figure of Merit (FOM) is defined as follows:

(4) $$FOM=P_{\mathrm{OUT}}\left(\mathrm{dBm}\right)+Gain\left(\mathrm{dB}\right)+20\log\left(f_{C}\left(\mathrm{GHz}\right)\right)+10\log\left(PAE\right)$$

Therefore, the proposed PA shows a competitive FOM regarding the high power at the antenna and higher PAE, while the supply voltage is kept lower than the other works, which makes it more suitable for low-voltage applications. Additionally, for short-range OTA WPT applications, the proposed PA satisfies the requirements and can be implemented in various arrays, such as 2-by-2 or 4-by-4 arrays, which can be used for the corresponding antenna arrays. Additionally, short-range OTA WPT applications operate as targets for high output efficiency with less supply, using the 5 GHz–6 GHz band. 

## 4. Conclusions

In this paper, design challenges and techniques to obtain a high efficiency for an OTA WPT transmitter are discussed, a PA is proposed, and the results are analyzed and compared with similar works in the literature. The PA was fabricated in a 180 nm CMOS process operating with a low supply voltage (1.8 V). A comparison between similar works and this work shows a good FOM value due to its high output power and efficiency with a lower supply voltage. To implement the PA, a balun with a high quality factor (Q) was designed, and the primary and secondary inductances were obtained to perform a suitable power matching. We conclude that the proposed PA is suitable for antenna-arrayed transmitters for power transmission, which is the objective of this work.

## Figures and Tables

**Table 1:** Performances Compared with Recent WPT PA.

Parameters	[13]	[14]	[15]	[16]	[17]	This Work
Technology	110 nm CMOS	180 nm CMOS	45 nm SOI-CMOS	110 nm CMOS	130 nm CMOS	180 nm CMOS
Supply Voltage (V)	2.6	3	4.8	2.3	2	1.8
Frequency (GHz)	9	10	12	9	12	5.8
Power Gian (dB)	11.6	25	9.8	10.6	17	20.05
Peak Output Power (dBm)	20.3	24.5	22.8	12.4	14	20.05
Peak PAE (%)	28.9	18	21.8	20.2	27	32.5
F.O.M.	245.59	262	247.56	235.13	245.63	250.48

## Data Availability

Not applicable.

## References

[1] Jang B., Hejazi A., Rad R.E., Qaragoez Y.M., Ali I., Pu Y., Hwang K.C., Yang Y., Lee K.-Y. (2021). A 15-W Triple-Mode Wireless Power Transmitting Unit with High System Efficiency Using Integrated Power Amplifier and DC–DC Converter. IEEE Trans. Ind. Electron..

[2] Valenta R., Durgin G.D. (2014). Harvesting Wireless Power: Survey of Energy-Harvester Conversion Efficiency in Far-Field, Wireless Power Transfer Systems. IEEE Microw. Mag..

[3] Rad R.E., Kim S., Rikan B.S., Lee K.-Y. A High Power High Efficient 5.8 GHz CMOS Class-A Power Amplifier for a WPT Application. Proceedings of the 2021 Twelfth International Conference on Ubiquitous and Future Networks (ICUFN).

[4] Hejazi A., Jang B., Rad R.E., Jo J., Rikan B.S., Pu Y., Yoo S.-S., Hwang K.C., Yang Y., Lee K.-Y. (2021). A 2.4 GHz Power Receiver Embedded with a Low-Power Transmitter and PCE of 53.8%, for Wireless Charging of IoT/Wearable Devices. IEEE Trans. Microw. Theory Tech..

[5] Kiani M. (2022). Wireless Power Transfer and Management for Medical Applications: Wireless power. IEEE Solid-State Circuits Mag..

[6] Meng M., Kiani M. (2017). A Hybrid Inductive-Ultrasonic Link for Wireless Power Transmission to Millimeter-Sized Biomedical Implants. IEEE Trans. Circuits Syst. II Express Briefs.

[7] Ibrahim A., Kiani M., Farajidavar A. A 64-channel wireless implantable system-on-chip for gastric electrical-wave recording. Proceedings of the 2016 IEEE Sensors.

[8] Springston C.S., Bao R., Farajidavar A. A 32-channel wireless system for recording gastric electrical activity. Proceedings of the 2016 38th Annual International Conference of the IEEE Engineering in Medicine and Biology Society (EMBC).

[9] Jia Y., Mirbozorgi A., Lee B., Khan W., Madi F.M., Weber A., Li W., Ghovanloo M. A mm-sized free-floating wirelessly powered implantable optical stimulating system-on-a-chip. Proceedings of the 2018 IEEE International Solid—State Circuits Conference—(ISSCC).

[10] Mirbozorgi S.A., Jia Y., Ghovanloo M. Power Efficiency and Power Delivery Measurement in Inductive Links with Arbitrary Source and Load Impedance Values. Proceedings of the 2018 IEEE Life Sciences Conference (LSC).

[11] Hajimiri A., Abiri B., Bohn F., Gal-Katziri M., Manohara M.H. (2021). Dynamic Focusing of Large Arrays for Wireless Power Transfer and Beyond. IEEE J. Solid-State Circuits.

[12] Bohn F., Abiri B., Hajimiri A. Fully integrated CMOS X-Band power amplifier quad with current reuse and dynamic digital feedback (DDF) capabilities. Proceedings of the 2017 IEEE Radio Frequency Integrated Circuits Symposium (RFIC).

[13] Park S., Jeon S. (2015). Wideband harmonic-tuned CMOS power amplifier with 19.5 dBm output power and 22.6% PAE over entire X-band. IET Electron. Lett..

[14] Comeau J.P., Thoenes E.W., Imhoff A., Morton M.A. X-Band +24dBm CMOS Power Amplifier with Transformer Power Combining. Proceedings of the 2011 IEEE 11th Topical Meeting on Silicon Monolithic Integrated Circuits in RF Systems.

[15] Chen J.-H., Helmi S.R., Jou A.Y.-S., Mohammadi S. (2013). A Wideband Power Amplifier in 45 nm CMOS SOI Technology for X Band Applications. IEEE Microwave Wirel. Comp. Lett..

[16] Park S., Jeon S. (2015). A full X-band CMOS amplifier with wideband class-E harmonic matching Microw. Opt. Technol. Lett..

[17] Park J., Park C. (2014). An X-band CMOS power amplifier with a driver stage using a shot-through current rejection technique. Microw. Opt. Technol. Lett..

